# Effectiveness and Tolerability of DOR/3TC/TDF in Experienced People with HIV Switching from RPV/FTC/TDF: A Retrospective, Single Center Cohort Study

**DOI:** 10.3390/ph17121706

**Published:** 2024-12-17

**Authors:** Stefania Cicalini, Simone Lanini, Roberta Gagliardini, Rita Bellagamba, Alessandra Vergori, Ilaria Mastrorosa, Valentina Mazzotta, Rozenn Esvan, Maria Maddalena Plazzi, Sandrine Ottou, Elisabetta Grilli, Federico De Zottis, Marisa Fusto, Jessica Paulicelli, Andrea Antinori

**Affiliations:** 1 Systemic and Immune Depression-Associated Infections Unit, Clinical Department, National Institute for Infectious Diseases Lazzaro Spallanzani IRCCS, 00149 Roma, Italy; stefania.cicalini@inmi.it; 2 Infectious Diseases Division, Department of Medicine, University of Udine and Azienda Sanitaria Universitaria Friuli Centrale, 33100 Udine, Italy; simone.lanini@uniud.it; 3 Viral Immunodeficiencies Unit, Clinical Department, National Institute for Infectious Diseases Lazzaro Spallanzani IRCCS, Via Portuense 292, 00149 Roma, Italy; rita.bellagamba@inmi.it (R.B.); alessandra.vergori@inmi.it (A.V.); ilaria.mastrorosa@inmi.it (I.M.); valentina.mazzotta@inmi.it (V.M.); rozenn.esvan@inmi.it (R.E.); maria.plazzi@inmi.it (M.M.P.); sandrine.ottou@inmi.it (S.O.); elisabetta.grilli@inmi.it (E.G.); federico.dezottis@inmi.it (F.D.Z.); marisa.fusto@inmi.it (M.F.); jessica.paulicelli@inmi.it (J.P.); andrea.antinori@inmi.it (A.A.)

**Keywords:** non-nucleoside reverse transcriptase inhibitors (NNRTIs), antiretroviral therapy optimization, single tablet regimen (STR)

## Abstract

**Background:** With advances in antiretroviral therapy for HIV treatment, newer drug combinations provide improved efficacy, safety, and compliance. This study evaluates switching to a regimen of doravirine (DOR), tenofovir disoproxil fumarate (TDF), and lamivudine (3TC) in a cohort of people living with HIV (PLWH). **Methods:** this Italian retrospective study included 426 PLWH who switched from rilpivirine (RPV)/TDF/emtricitabine (FTC) to DOR/3TC/TDF. The analysis focused on treatment effectiveness, safety, and metabolic and renal markers. **Results:** this study reports a treatment failure (defined as virological failure or discontinuation of the regimen) rate of 2.34% (95% confidence interval, 1.28–4.50%), with significant improvement in CD4 counts (+49.93 cells/µL, *p* < 0.001). Notably, the switch to DOR/3TC/TDF did not result in adverse metabolic effects or significant changes in renal function. Analysis of lipid profiles showed stabilization in the majority of PLWH. **Conclusions:** this study indicates that switching to a DOR/3TC/TDF from RPV/TDF/FTC is an effective and well-tolerated option for PLWH, with benefits in terms of maintaining viral suppression, CD4 count recovery, and metabolic health, without evidence of renal impairment. These results support the continued use of DOR/3TC/TDF as part of HIV treatment strategies and highlight the need for ongoing research to refine ART regimens for different populations.

## 1. Introduction

In the current HIV treatment landscape, prioritizing antiretroviral therapy (ART) regimens that not only effectively suppress viral load but also minimize long-term side effects has become fundamental.

Indeed, given the need for lifelong treatment, it remains essential to evaluate the durability of virologic response and the long-term safety and tolerability of ART regimens. In particular, the metabolic effects of an ART regimen are critical to improving the quality of life and long-term health outcomes of people living with HIV (PLWH), who are at increased risk of cardiovascular diseases and metabolic syndrome due to both the virus and antiretroviral drugs [1,2,3,4]. Currently, ART is recommended for all PLWH, irrespective of CD4 counts [5]. The most common ARTs prescribed worldwide are three- or two-drug regimens composed of integrase inhibitors, protease inhibitors, non-nucleoside reverse transcriptase inhibitors (NNRTIs), and nucleoside reverse transcriptase inhibitors (NRTI). When selecting the best treatment plan, it should be taken into account factors such as existing health conditions, current medications, and any resistance to antiretroviral drugs.

Doravirine (DOR) is the latest approved antiretroviral of the class of non-nucleoside reverse transcriptase inhibitors (NNRTIs). It is recommended in combination with lamivudine (3TC) or emtricitabine and tenofovir disoproxil fumarate (TDF) as a first line and as a switch regimen for adults with HIV-1 [5].

Data from clinical trials and real-world studies have demonstrated the long-term efficacy and safety profile of DOR in terms of virological control and metabolic profile, in both ART-naïve and experienced PLWH [6,7,8,9,10,11,12,13,14], showing minimal impact on lipid levels, body weight, and renal function, despite the combination with TDF in most cases.

Moreover, DOR has a low potential for clinically meaningful drug–drug interactions, can be administered without food restrictions, and presents a different genotypic resistance profile from the other drugs of NNRTI class, especially from rilpivirine [15]. DOR is available as a single drug that can be combined with other antiretroviral drugs and as a single tablet formulation combined with 3TC and TDF. The combination of DOR/3TC/TDF in a single tablet could offer significant advantages, particularly because of its potential to address the complex needs of PLWH, with a focus on efficacy, metabolic health, and improved adherence to lifelong treatment. Moreover, co-administration with TDF may raise concerns about the feasibility of this regimen in people at risk or affected by renal and bone comorbidities [16,17].

Here, we present the results of a large historical cohort study involving ART-experienced PLWH to evaluate the potential advantages of DOR in PLWH who switched from RPV without changing their NRTI backbone, in terms of virological response, immunological parameters, and potential changes in metabolic biomarkers.

## 2. Results

### 2.1. Descriptive Analysis

**Figure 1:**
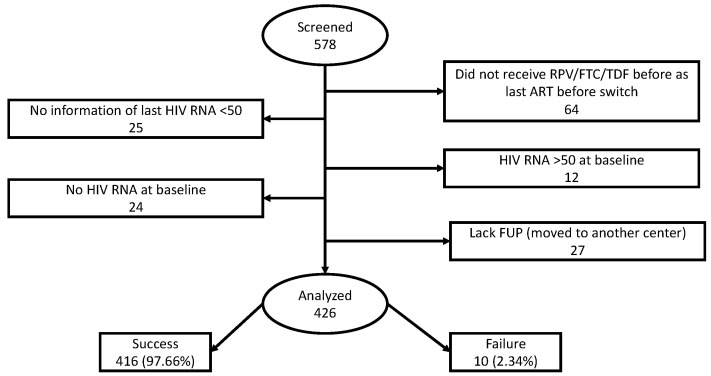
Sample selection and main outcome.

Between 1 January 2020 and 1 April 2022, a total of 578 PLWH switched to DOR/3TC/TDF in clinical practice. Of these, 64 were excluded because they did not receive RPV/FTC/TDF as last ART regimen, 12 were not included in the analysis because HIV-RNA was higher than 50 copies/mL, 24 because information on HIV-RNA at switch was missing, 25 because information on the last detectable HIV-RNA was not available, and 27 due to lack of follow up (FU) (Figure 1).

Thus, the remaining 426 PLWH were included in the analysis. Table 1 shows the main demographic, viro-immunological, and metabolic characteristics of PLWH at the time of switching. Their median age was 47 years (interquartile range, IQR 38–54), and 296 (69.48%) of them were males.

The main route of HIV transmission was through heterosexual intercourse (*n* = 291; 68.31%), followed by sex between men (*n* = 93; 21.83%) and injecting drug use (*n* = 36; 8.45%). Non-Caucasian people were 63 (14.79%) of the sample, 96 (22.54%) PLWH had a CD4 nadir below 200 cells/mm^3^ and 36 (8.45%) had a previous AIDS diagnosis. They were virologically suppressed for 6 years in median (IQR 4–10). At the time of switching, all of them were virologically suppressed; median CD4 cell count was 719 (IQR 574–903) with a median CD4/CD8 ratio of 1.02 (IQR 0.76–1.35). Median creatinine was 0.95 (IQR 0.81–1.04).

### 2.2. Primary Outcome: Treatment Failure

Ten individuals met the definition of treatment failure, with a cumulative risk of 2.34% (95% confidence interval, CI, 1.28–4.50%). Of these, six met the definition for virologic failure and four discontinued DOR/3TC/TDF due to clinical decision. Virological failure includes six individuals with HIV-RNA > 200 copies/mL. Among them, four reported poor adherence and continued DOR/3TC/TDF with improved viral control (three had HIV-RNA < 50 copies/mL at the next visit and one had a viral decline from 51,778 cp/mL to 983 cp/mL), one had HIV-RNA > 200 copies/mL at month 12, and one regained viral control as a result of switching to a PI-based regimen. Historical genotypes pre-switch did not show resistance mutations for NRTI and NNRTI for five out of six of these PLWH, while one PLWH did not have any previous resistance data available. Only in one subject a new genotypic resistance test was performed during virological failure with DOR/3TC/TDF, and this did not detect relevant mutations.

In the four individuals who stopped DOR/3TC/TDF due to clinician’s decision, the main reason was concern about potential renal safety for three cases with significant risk of renal damage but normal creatinine levels (insulin resistance leading to diabetes with glycosuria and severe prostatic hypertrophy with recurrent cystitis) and for detection of osteopenia in the Dxa scan in the remaining one individual.

### 2.3. Virologic Response

**Figure 2:**
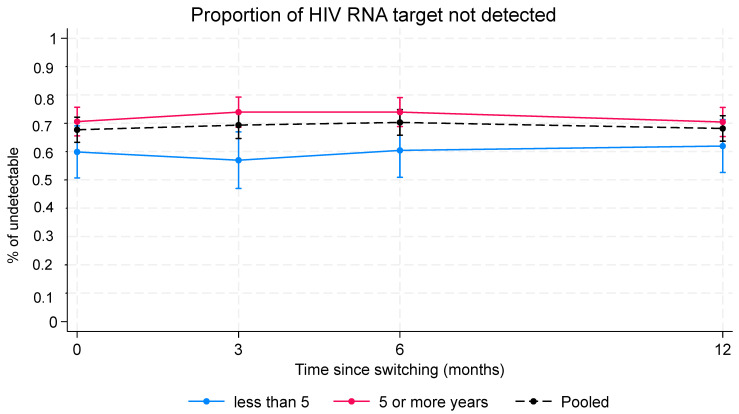
Proportion of TND PLWH. Analysis was performed on a set of 461 PLWH, accounting for a total of 1693 HIV-RNA measurements. Estimates were calculated using a mixed-effects logistic regression model with a random intercept at the individual level. In red, PLWH with more than 5 years of virologic control; in blue, PLWH who achieved HIV RNA < 50 copies/mL for 5 years or less. Within-group analysis: estimates for PLWH with more than 5 years of virologic control and those who achieved HIV-RNA < 50 copies/mL for 5 years or less over time. Between-group analysis: comparison between PLWH with more than 5 years of virologic control and those who achieved HIV RNA < 50 copies/mL for 5 years or less at each follow-up time point.

The analysis of PLWH with complete virologic control (target not detected, TND) included 416 individuals, for a total of 1530 HIV-RNA measurements (mean measurements for each individual 3.7; range 2–4). Complete virologic response for each FU endpoint was calculated independently for PLWH who were virologically controlled for more than 5 years (*n* = 302) and for those who had HIV-RNA < 50 copies/mL for 5 years or less (*n* = 114), and then were simultaneously adjusted for sex, age, and CD4 nadir (Figure 2 and Supplementary Materials).

The analysis shows that the proportion of TND was significantly higher in PLWH who were virologically suppressed for more than 5 years compared with those who were virologically suppressed for 5 years or less (Figure 2; *p* = 0.019). The proportion of TND remained consistently above 70% throughout observations in the group of PLWH who were virologically suppressed for more than 5 years (within-group time analysis *p* = 0.858) and around 60% in the others (within-group time analysis *p* = 0.507).

We also found that male individuals (odds ratio, OR, 0.58; 95% CI 0.38–0.93; *p* = 0.025) and those with CD4 nadir below 200 cells/µL (OR 0.57, 95% CI 0.34–0.95 *p* = 0.032) were less likely to be undetectable than female individuals and those with a CD4 nadir above 200 cells/µL. There was no association between age and the proportion of undetectable PLWH (*p* = 0.424).

### 2.4. Variation In Absolute Count of CD4 Lymphocytes

Analyses of variation in CD4 cell count included 416 PLWH for a total of 1377 CD4 measurements (mean measurements for each individual 3.2; range 1–4). Estimates of mean CD4 change at each FU endpoint was calculated independently for the PLWH who were virologically suppressed for more than 5 years (*n* = 302) and for those who were virologically suppressed for 5 years or less (*n* = 114), and then were simultaneously adjusted for sex, age, and CD4 nadir.

**Figure 3:**
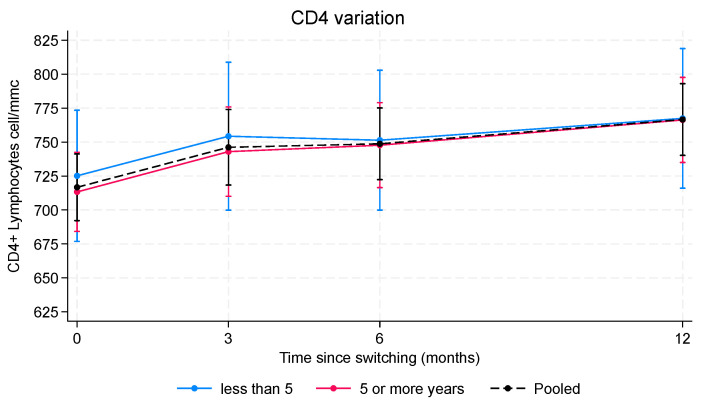
CD4 lymphocyte variation over time. The analysis was performed on a set of 461 PLWH with a total of 1693 HIV RNA measurements. Estimates were calculated using a mixed effects linear regression model with a random intercept at the individual level. In red, PLWH with more than 5 years of virologic control; in blue, PLWH who achieved HIV-RNA < 50 copies/mL for 5 years or less. Black dotted line shows pooled estimates for both groups. Within-group analysis: estimates for PLWH with more than 5 years of virologic control and those who achieved HIV-RNA < 50 copies/mL for 5 years or less over time. Between-group analysis: comparison between PLWH with more than 5 years of virologic control and those who achieved HIV RNA < 50 copies/mL for 5 years or less at each follow-up endpoint.

Analysis of variation in CD4 count showed a significant increase over time in both groups (Figure 3 and Supplementary Materials).

In PLWH with HIV-RNA < 50 copies/mL for less than 5 years, mean CD4 counts increased from 725.16 cells/µL (SE 24.63) to 767.43 cells/µL (SE 26.22) over 1 year of follow-up, with a significant mean increase of 42.27 cells/µL (95% CI 8.60–75.94; *p* = 0.010). Similarly, in those who were virologically suppressed for more than 5 years, mean CD4 counts increased from 713.35 cells/µL (SE 14.84) to 766.30 cells/µL (SE 15.89), with a significant mean increase of 52.96 cells/µL (95% CI 32.22–73.70; *p* < 0.001).

We found no significant differences in mean CD4 counts between the groups at any FU time (*p* = 0.966). Overall observed mean variation in CD4 in the pooled group increased from 716.69 cells/µL (SE 12.54) to 766.62 cells/µL (SE 13.46), with a significant mean increase of 49.93 cells/µL (95% CI 31.07–68.79; *p* < 0.001).

Multivariable analyses also show a significant inverse association between mean CD4 counts and CD4 nadir less than 200 cells/µL (mean reduction −104.58, 95% CI −158.58 to −50.59; *p* < 0.001). We found no significant association between mean CD4 count and either sex (*p* = 0.097) or age (*p* = 0.103).

### 2.5. Variation In CD4/CD8 Ratio

Analyses of variation in CD4/CD8 ratio included 412 PLWH for a total of 1317 CD4 and CD8 measurements (mean measurements for each individual 3.2; range 1–4). Estimates of the mean CD4/CD8 ratio change at each FU endpoint were calculated independently for the PLWH who were virologically suppressed for more than 5 years (*n* = 298) and for those who were virologically suppressed for 5 years or less (*n* = 114), and then were simultaneously adjusted for sex, age, and CD4 nadir.

**Figure 4:**
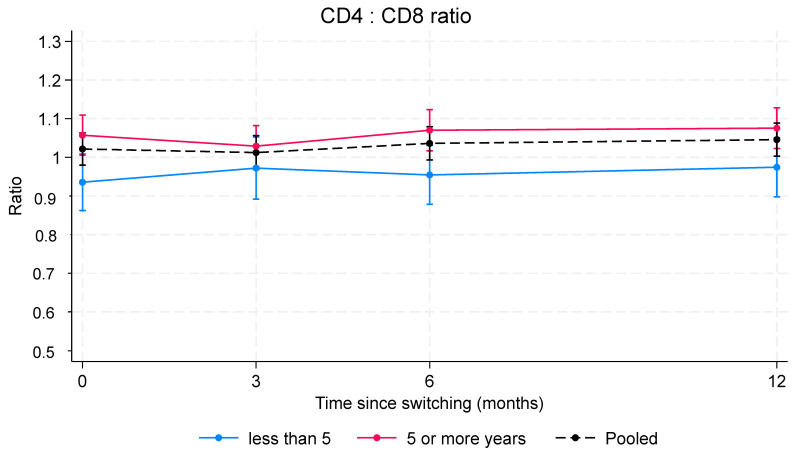
Change in CD4:CD8 ratio over time. The analysis was performed on a set of 461 PLWH with a total of 1693 HIV-RNA measurements. Estimates were calculated using a mixed effects linear regression model with a random intercept at the individual level. In red, PLWH with more than 5 years of virologic control; in blue, PLWH who achieved HIV-RNA < 50 copies/mL for 5 years or less. Within-group analysis: estimates for PLWH with more than 5 years of virologic control and those who achieved HIV-RNA < 50 copies/mL for 5 years or less over time. Between-group analysis: comparison between PLWH with more than 5 years of virologic control and those who achieved HIV-RNA < 50 copies/mL for 5 years or less at each follow-up endpoint.

The analysis for variation in CD4/CD8 ratio (Figure 4) showed an increasing trend in CD4/CD8 ratio over time in PLWH who were virologically suppressed for less than 5 years. In these subjects, the mean CD4/CD8 ratio increased over time from 0.94 (SE 0.04) to 0.97 (SE 0.04), with a mean increase of 0.04 (95% CI 0.00–0.08; *p*-value 0.061).

No significant change in CD4/CD8 ratio was observed in PLWH who were virologically suppressed for more than 5 years. In these PLWH, the CD4/CD8 ratio was consistently above 1, with minimal fluctuation (*p* = 0.290). The between-group analysis showed, as expected, that the CD4/CD8 ratio was significantly higher in PLWH who had been virologically controlled for more than 5 years than in those with HIV-RNA < 50 copies/mL for less than 5 years (*p* = 0.017). Multivariable analyses also showed a significant inverse association between mean CD4/CD8 ratio values and either CD4 nadir less than 200 cells/µL (mean reduction −0.16%; 95% CI −0.25 to −0.07; *p* < 0.001) and sex (mean reduction in men −0.28; 95% CI −0.38 to −0.18; *p* < 0.001). We found no significant association between CD4/CD8 ratio and age (*p* = 0.167).

### 2.6. Safety and Metabolic Parameters

Analyses for safety and metabolic parameters were performed on all available measures for the 416 individuals who were retained for the full duration of FU. We found no clinically significant difference on all the parameters that were analyzed, as reported in Table 2.

## 3. Discussion

Consistent with data from randomized clinical trials and observational studies [6,7,8,9,10,11,12,13,14,18,19], this real-world study demonstrated the effectiveness of the DOR/3TC/TDF regimen in maintaining virologic suppression in ART-experienced PLWH, with a low 1-year cumulative risk of treatment failure of 2.34%.

In addition, a closer look at the ten PLWH who met our treatment failure definition provides a more nuanced view of regimen performance and PLWH management. Of these, six experienced virologic failure, five of whom were attributed to poor compliance; however, subsequent continuation of therapy resulted in improved virologic control for most. Only one subject had a confirmed virologic failure without recovery on the same regimen, requiring a switch to a PI-based regimen. In the four subjects who discontinued the regimen due to a clinical decision, the reasons were potential renal safety concerns in three cases, and one case was related to osteopenia. Our rate of treatment failure was lower than what reported in another large retrospective study on doravirine-based ART by Oomen et al. [20], where treatment failure occurred in 22.9% of PLWH at 2 years, mainly because of switching to other regimens in conditions of virological suppression.

This study demonstrates, other than the maintenance of viral suppression, also a small but statistically significant immunological improvement of about 50 CD4 cells per mL over the first year of follow-up. Indeed, the significant overall increase in CD4 counts and the slight increase in the CD4/CD8 ratio in PLWH with less than five years of viral suppression suggests that switching from RPV to DOR could allow for immunological restoration. Indeed, the different pattern of CD4 recovery, which occurred immediately after switching in PLWH with more than 5 years of viral control, provides preliminary evidence of immunological restoration with this drug combination, which will need to be confirmed in future studies. Notably, this potential effect of DOR on CD4 count has been recently reported in other real-world experiences in Europe [21,22]. However, it has to be noted that our study lacked a control group, and this potential improvement in CD4 cells count was not documented in other real-world studies and in clinical trials [9,14,20].

Another important contribution of the present study is the detailed evaluation of the metabolic and renal safety profile of the DOR/3TC/TDF regimen. Given the chronic nature of HIV treatment and the need for lifelong ART, concerns about long-term adverse effects, including renal dysfunction and metabolic abnormalities, are paramount. The results of this study suggest that the DOR/3TC/TDF regimen is well tolerated and does not cause significant adverse changes in metabolic markers and confirm the favorable results of randomized trials and of real-world settings [9,14,22,23,24]. Because reductions in renal function are possibly observed in PLWH in therapy, it is important to understand the long-term effects of a DOR-based regimen on renal function, especially given its combination with TDF. The absence of pharmacokinetic boosters, such as ritonavir or cobicistat in the DOR/3TC/TDF combination, and the low impact of DOR on safety parameters potentially mitigates the nephrotoxic risks commonly associated with TDF, providing a safer renal profile for PLWH [16,17].

No change in lipid profile nor in body weight was observed, although all our PLWH were already on TDF-containing regimens. Moreover, this study is one of the largest retrospective reports about doravirine use in real-world settings.

Although this study provides valuable evidence, several limitations must be acknowledged. The historical design, although useful for examining real-world outcomes, is subject to the biases associated with retrospective data collection and the lack of a control group for direct comparison. In addition, the single-center setting of the study, albeit with a large sample size, may limit the generalizability of the findings to other populations and settings. Moreover, this study has been designed to provide information about the switch from RPV/TDF/FTC to DOR/3TC/TDF, and thus it cannot inform on potential impact of switching to DOR/3TC/TDF from other ART, e.g., INSTI, or TAF-containing ARTs.

Future research should include randomized controlled trials and multi-center studies to validate these findings and to investigate the efficacy and safety of the regimen in different populations and geographical settings. Another area for future research is the long-term impact of the DOR/3TC/TDF regimen on PLWH-reported outcomes, including quality of life, treatment adherence, and, most importantly, bone turnover, which could not be collected in this study. Understanding these aspects can help to outline the practical benefits and challenges of the regimen, supporting patient-centered care strategies and informing ART regimen selection.

Despite these limitations, our results have several interesting clinical implications. Firstly, they support the consideration of the DOR/3TC/TDF as an effective and safe regimen in the switching strategy in PLWH virologically suppressed. Safety profiles may support the concern of the still-current feasibility of using TDF as nucleoside backbone when doravirine was given. Secondly, it could pave the way for further studies to assess the potential impact of DOR/3TC/TDF in selected individuals with good renal function but potentially increased risk of metabolic impairment. Finally, the effectiveness and favorable tolerability profile may help to consider this regimen valid from a cost-effectiveness perspective. These assumptions, even in the current scenario of the dominant integrase-inhibitors drug class, align with the confirmed position of recommended ART regimen of DOR/3TC/TDF in the EACS Guidelines [5].

In conclusion, this study adds to the growing body of evidence supporting the use of the DOR/3TC/TDF regimen in the treatment of HIV. By demonstrating a balance between efficacy and tolerability, particularly regarding metabolic and renal health, the regimen represents a relevant option for PLWH.

## 4. Materials and Methods

### 4.1. Study Design

This retrospective cohort study was conducted at the National Institute for Infectious Diseases (INMI) Lazzaro Spallanzani in Rome, Italy, a leading research and clinical care center for infectious diseases. This study aimed to explore the effectiveness and safety of switching from a RPV-based regimen to a DOR-based regimen in real-life. The study population (P) includes PLWH who switched form RPV/FTC/TDF to DOR/3TC/TDF (I). To minimize the potential issues associated with the retrospective design of the study, we chose a solid unambiguous primary binary endpoint (O) such as therapy failure (see below for definition). As for other explorative studies, we did not include a formal a priori comparator group (C). However, comparative analyses were carried out on analyses’ driven questions that may be relevant for further interpretation of our results. This clinical choice of switching was guided by several key factors that underscored the advantages of DOR over RPV. Notably, DOR has shown strong virological efficacy, even in PLWH with higher baseline viral loads, where RPV’s effectiveness may be compromised. Additionally, DOR is associated with fewer drug–drug interactions and does not require food for optimal absorption, making it a more suitable option for patients on complex medication regimens or those with co-morbid conditions.

### 4.2. Participants and Data Collection

This study was designed as a historical cohort of adult individuals (aged ≥ 18 years) with HIV-1 infection and HIV-RNA < 50 copies/mL at time of the switch, who switched from a RPV/FTC/TDF to a DOR/3TC/TDF regimen in clinical practice between 1 January 2020 and 1 April 2022. Clinical information was collected by reviewing medical records. This study was performed in accordance with the 1964 Declaration of Helsinki, and later amendments and approved by the ethics committee. PLWH signed an informed consent form for use of their clinical and laboratory data using an aggregated and pseudo-anonymous form. We excluded from the analyses PLWH who started follow-up at INMI when HIV-RNA was already <50 copies/mL; PLWH who had no information about the time of last detectable HIV-RNA; PLWH who received DOR/3TC/TDF but had HIV-RNA ≥ 50 copies/mL at the time of the switch; and PLWH who received DOR/3TC/TDF with less than 3 months of follow up after switching.

### 4.3. Variables

We reviewed charts and analyzed routinely collected data, which were anonymized by coding all personal identifiers. Demographic, epidemiological, clinical, and laboratory information routinely collected (HIV-RNA, CD4 cells count, CD4/CD8 ratio, ALT, AST, LDL, HDL, total cholesterol, triglycerides, creatinine, glycemia, and body weight) was retrieved. The primary endpoint was the cumulative probability of treatment failure, a composite outcome that included virological failure (defined as confirmed HIV-RNA > 50 copies/mL or one single determination > 200 copies/mL) or discontinuation of the regimen.

Secondary endpoints were measured only in PLWH without treatment failure and were designed to evaluate the temporal variation in immunological and metabolic markers, including CD4 cell count, CD4/CD8 ratio, ALT, AST, LDL, HDL, total cholesterol, triglycerides, creatinine, fasting glycemia, and body weight. In addition, we assessed the proportion of PLWH with target not detected, defined as undetectable HIV-RNA versus those with target detected, defined as HIV-RNA detectable at any level. Plasma HIV-1-RNA was evaluated with the Aptima HIV-1 Quant Dx Assay (Hologic, Inc., San Diego, CA, USA), which reports quantitative HIV-RNA results from 30 to 10,000,000 copies/mL.

### 4.4. Sample Size and Statistical Analysis

We analyzed all eligible PLWH who switched from RPV/FTC/TDF to DOR/3TC/TDF while on virological suppression. Due to the explorative nature of the analyses with no primary hypothesis and the single-arm retrospective study design, no formal sample size calculation was performed. Descriptive analysis was performed by reporting the distribution of PLWH’s characteristics by proportions (categorical variables) or median and interquartile range (continuous variables) on the whole sample. The cumulative risk of failure was calculated using a standard logistic regression model.

The analysis of TND was conducted using a mixed logistic regression model, chosen for its robustness in modeling variations in repeated measures that are inherently correlated. This approach allowed us to model the proportion of TND (a binary variable) over time following the treatment switch. The model included the following components: (a) a random intercept at the patient level to account for the correlation of repeated measures from the same patient; (b) TND as the binary dependent variable; (c) time during follow-up (a categorical 4-level primary predictor) to model the variation in TND proportions over time; (d) time of the last detectable HIV-RNA (>50 copies/mL, a 2-level categorical variable: ≤5 years or >5 years), included as a potential effect modifier; (e) full categorical interaction between follow-up time and the last detectable HIV-RNA, to account for effect modification; (f) a set of additional potential confounders, including age (continuous variable), sex (binary variable), and CD4 cell nadir (binary variable, ≤200 cells/mm^3^ or >200 cells/mm^3^).

A similar approach was applied to assess variations in immunological parameters, including CD4 counts and the CD4/CD8 ratio. Two separate mixed linear regression models were used with the following structure: (a) a random intercept at the patient level to address the correlation of repeated measures; (b) either CD4 count or CD4/CD8 ratio as the dependent variable; (c) time during follow-up (categorical 4-level primary predictor); (d) time of the last detectable HIV-RNA (>50 copies/mL, a 2-level categorical variable: ≤5 years or >5 years) as a potential effect modifier; (e) full categorical interaction between follow-up time and the last detectable HIV-RNA to account for effect modification; and (f) additional potential confounders, including age (continuous variable), sex (binary variable), and CD4 cell nadir (binary variable, ≤200 cells/mm^3^ or >200 cells/mm^3^). To address potential heteroscedasticity, analyses for CD4 count and CD4/CD8 ratio were conducted using natural log-transformed dependent variables, with estimates back-transformed for improved interpretation. Adjustments for multiple comparisons were made using the Bonferroni method.

Finally, variations in metabolic parameters, including total cholesterol (mg/dL), HDL (mg/dL), LDL (mg/dL), triglycerides (mg/dL), ALT (U/L), AST (U/L), glycemia (mg/dL), and body weight (kg), were analyzed using a mixed linear regression model with a random intercept at the patient level. This model assessed mean differences between baseline (enrollment) and the end of follow-up. The analysis was censored after 1 year from the switch to DOR/3TC/TDF or at treatment failure, whichever occurred first.

## Figures and Tables

**Table 1:** Main characteristics of PLWH at switch to DOR/3TC/TDF.

PLWH Characteristics (*n* = 426)	
Median age in years (IQR)	47 (38–54)
Previous AIDS diagnosis, *n* (%)	36 (8.45%)
HCV-Ab positive, *n* (%)	56 (13.15%)
HBsAg positive, *n* (%)	42 (9.86%)
Male sex, *n* (%)	296 (69.48%)
Mode of HIV Transmission, *n* (%)	
Heterosexual transmission Men who have sex with men Injection drug users Other	291 (68.31%) 93 (21.83%) 36 (8.45%) 6(1.41%)
Non-Caucasian ethnicity, *n* (%)	63 (14.79%)
CD4 nadir less than 200 cell/mmc, *n* (%)	96 (22.54%)
Median creatinine (IQR)	0.95 (0.81–1.04)
Median HDL (IQR)	43 (36–51)
Median LDL (IQR)	114 (95–141)
Median total cholesterol (IQR)	171 (146.5–195)
Median triglycerides (IQR)	93 (68–135.5)
Median CD4 (IQR)	719 (574–903)
Median CD4/CD8 ratio (IQR)	1.02 (0.76–1.35)
Median ALT (IQR)	24 (17–34)
Median AST (IQR)	25 (20–33)
Median glycemia (IQR)	82 (76–90)
Median time since last HIV RNA > 50 cp/mL in years (IQR)	6 (4–10)
Median time since first HIV positive test in years (IQR)	10 (6–14)

IQR = interquartile range; *n* = number of PLWH.

**Table 2:** Variation in biochemical parameters between enrollment and the end of FU.

Parameters	Average Enrollment Value	Average Change at the End of FU				PLWH, *n*	Measures
		Variation	95% CI		*p*-Value		
Creatinine (mg/dL)	0.93	−0.01	−0.02	0.00	0.118	414	804
Total cholesterol (mg/dL)	170.25	2.33	−0.37	5.03	0.091	414	788
HDL (mg/dL)	45.55	0.79	−0.12	1.69	0.089	414	776
LDL (mg/dL)	116.60	1.29	−1.01	3.60	0.272	413	778
Triglycerides (mg/dL)	108.71	−0.91	−6.04	4.22	0.729	412	776
ALT (U/L)	30.06	−1.82	−4.34	0.70	0.157	407	767
AST (U/L)	29.07	−1.92	−3.92	0.09	0.065	407	767
Glycemia (mg/dL)	85.24	1.37	−1.13	3.87	0.284	415	764
Body weight (Kg)	73.71	−0.18	−0.52	0.16	0.299	350	700

FU, follow up; CI, confidence interval; PLWH, people living with HIV.

## Data Availability

The datasets generated during the current study are not publicly available. Appropriate agreement of data sharing can be arranged after a reasonable request to the corresponding author.

## Supplementary Materials

The following supporting information can be downloaded at: https://www.mdpi.com/article/10.3390/ph17121706/s1.
