# Supplementary material for: Effectiveness and Tolerability of DOR/3TC/TDF in Experienced People with HIV Switching from RPV/FTC/TDF: A Retrospective, Single Center Cohort Study
Source: Pharmaceuticals (Basel). 2024 Dec 17;17(12):1706. doi: 10.3390/ph17121706 (PMC11676077; doi:10.3390/ph17121706)
Supplement: Supplementary file 1 [file pharmaceuticals-17-01706-s001.zip › pharmaceuticals-3312747-supplementary.pdf]

Supplementary materials

Virologic response- target not detected (TND) analysis

FU time, months	Undetectable for more than 5 years (N=302)				Undetectable for 5 years or less (N=114)				Comparison between groups			
	estimate	95% CI		p	estimate	95% CI		p	estimate	95% CI		p
0	70.58%	65.53%	75.62%	0.858	59.87%	50.67%	69.07%	0.507	10.90%	-2.79%	24.60%	0.019
3	73.92%	68.61%	79.23%		56.93%	46.94%	66.92%		17.30%	2.54%	32.06%	
6	73.92%	68.81%	79.03%		60.41%	50.88%	69.94%		13.76%	-0.35%	27.86%	
12	70.43%	65.28%	75.59%		61.95%	52.62%	71.28%		8.64%	-5.26%	22.54%	

Variation of absolute count of CD4 lymphocytes

FU time, months	Within-groups analyses												Between gruop difference
	Undetectable for more than 5 years (N=302)						Undetectable for 5 years or less (N=114)						
	CD4 counts (cell/mmc)		CD4 variation (cell/mmc)				CD4 counts (cell/mmc)		CD4 variation (cell/mmc)				
	Mean	SE	Mean	95% CI		p	Mean	SE	Mean	95% CI		p	p
0	713.35	14.84	-	-	-	-	725.16	24.63	-	-	-	-	0.966
3	742.94	16.77	29.59	4.78	54.41	0.015	754.29	27.81	29.13	-11.57	69.84	0.217	
6	747.71	15.95	34.37	12.72	56.01	0.001	751.40	26.27	26.24	-8.94	61.42	0.189	
12	766.30	15.98	52.96	32.22	73.70	<0.001	767.43	26.22	42.27	8.60	75.94	0.010	

Variation of CD4/CD8 ratio over time

FU time, months	Within-groups analyses												Between group difference
	Undetectable for more than 5 years (N=298)						Undetectable for 5 years or less (N=114)						
	CD4/CD8 Ratio		CD4/CD8 Ratio (difference)				CD4/CD8 Ratio		CD4/CD8 Ratio (difference)				
	Mean	SE	Mean	95% CI		p	Mean	SE	Mean	95% CI		p	p
0	1.06	0.03	-	-	-	-	0.94	0.04	-	-	-	-	0.017
3	1.03	0.03	-0.03	-0.06	0.00	0.110	0.97	0.04	0.04	-0.01	0.09	0.187	
6	1.07	0.03	0.01	-0.02	0.04	0.660	0.95	0.04	0.02	-0.02	0.06	0.623	
12	1.08	0.03	0.02	-0.01	0.05	0.290	0.97	0.04	0.04	0.00	0.08	0.061	
